# Current sampling and sequencing biases of Lassa mammarenavirus limit inference from phylogeography and molecular epidemiology in Lassa fever endemic regions

**DOI:** 10.1371/journal.pgph.0002159

**Published:** 2023-11-08

**Authors:** Liã Bárbara Arruda, Hayley Beth Free, David Simons, Rashid Ansumana, Linzy Elton, Najmul Haider, Isobella Honeyborne, Danny Asogun, Timothy D. McHugh, Francine Ntoumi, Alimuddin Zumla, Richard Kock

**Affiliations:** 1 Division of Infection and Immunity, Centre for Clinical Microbiology, University College London, London, United Kingdom; 2 The Royal Veterinary College, University of London, Hatfield, United Kingdom; 3 School of Community Health Sciences, Njala University, Bo, Sierra Leone; 4 Ekpoma and Irrua Specialist Teaching Hospital, Ambrose Alli University, Irrua, Nigeria; 5 Fondation Congolaise Pour la Recherche Médicale (FCRM), Brazzaville, Republic of Congo; 6 Institute for Tropical Medicine, University of Tübingen, Tübingen, Germany; 7 NIHR Biomedical Research Centre, UCL Hospitals NHS Foundation Trust, London, United Kingdom; University of Hong Kong, HONG KONG

## Abstract

Lassa fever (LF) is a potentially lethal viral haemorrhagic infection of humans caused by *Lassa mammarenavirus* (LASV). It is an important endemic zoonotic disease in West Africa with growing evidence for increasing frequency and sizes of outbreaks. Phylogeographic and molecular epidemiology methods have projected expansion of the Lassa fever endemic zone in the context of future global change. The Natal multimammate mouse (*Mastomys natalensis*) is the predominant LASV reservoir, with few studies investigating the role of other animal species. To explore host sequencing biases, all LASV nucleotide sequences and associated metadata available on GenBank (n = 2,298) were retrieved. Most data originated from Nigeria (54%), Guinea (20%) and Sierra Leone (14%). Data from non-human hosts (n = 703) were limited and only 69 sequences encompassed complete genes. We found a strong positive correlation between the number of confirmed human cases and sequences at the country level (*r* = 0.93 (95% Confidence Interval = 0.71–0.98), *p* < 0.001) but no correlation exists between confirmed cases and the number of available rodent sequences (*r* = -0.019 (95% C.I. -0.71–0.69), *p =* 0.96). Spatial modelling of sequencing effort highlighted current biases in locations of available sequences, with increased sequencing effort observed in Southern Guinea and Southern Nigeria. Phylogenetic analyses showed geographic clustering of LASV lineages, suggestive of isolated events of human-to-rodent transmission and the emergence of currently circulating strains of LASV from the year 1498 in Nigeria. Overall, the current study highlights significant geographic limitations in LASV surveillance, particularly, in non-human hosts. Further investigation of the non-human reservoir of LASV, alongside expanded surveillance, are required for precise characterisation of the emergence and dispersal of LASV. Accurate surveillance of LASV circulation in non-human hosts is vital to guide early detection and initiation of public health interventions for future Lassa fever outbreaks.

## 1 Introduction

Lassa fever (LF) is a lethal zoonotic viral haemorrhagic disease of humans, caused by *Lassa mammarenavirus* (LASV). The estimated westward route of dispersal of the seven lineages of this arenavirus has been used to project the potential for LF to extend beyond the current endemic zone [1], currently concentrated into eight West African countries: Benin, Ghana, Guinea, Liberia, Mali, Sierra Leone, Togo and Nigeria (S1 Fig) [2].

Epidemiological data on LF is limited and constrained by availability of current testing and reporting in the endemic region, making accurate estimates of its true burden challenging [3]. Many individuals infected with LASV do not seek healthcare with up to 80% of infections assumed asymptomatic or presenting as mild illness [4]. Estimates based on longitudinal serological surveys in Sierra Leone in the early 1980’s indicated that 100,000 to 300,000 infections of LF occurred annually in West Africa, with more recent estimates being up to 900,000 infections/year [4,5]. Identification of symptomatic cases is further confounded by overlapping symptoms with other diseases (e.g., malaria) and lack of available diagnostic methods [6–9].

Although human-to-human transmission of LF has been reported–typically associated with nosocomial outbreaks–these are rare events when compared with spillover from rodent hosts [10]. Humans become infected with LASV upon contact with or inhalation of excretions from the rodent species [11,12]. The Natal multimammate mouse (*Mastomys natalensis*) is considered the primary reservoir of LASV. Despite 11 other rodent species having been found to be acutely infected or have seropositivity to LASV including; *Mastomys erythroleucus*, *Hylomyscus pamfi*, *Mus baoulei* and *Rattus rattus* there are a limited number of studies investigating the role in LASV transmission in these other rodents [13–18].

Access to diagnostic tests varies spatially, and the increased availability at centres of excellence in LF treatment and research such as the Irrua Specialist Teaching Hospital, Nigeria and Kenema General Hospital, Sierra Leone results in a spatial bias of reported cases from these locations [19]. This results in uneven LF surveillance initiatives across host species and endemic regions. The consequent paucity of LASV genomic data and associated metadata leads to spatial biases playing a potentially important role in understanding of the disease epidemiology and ecology. In order to identify surveillance gaps and quantify some of the current biases in genomic surveillance, we performed a study of LASV nucleotide sequences available from the National Centre for Biotechnology Information (NCBI) GenBank, using associated metadata to spatially model sequencing effort, adjusted for the number of suspected and confirmed human LF cases to determine potential biases in locations of available sequences or significant geographic limitations in LASV surveillance, particularly, in non-human hosts.

## 2 Methods

### 2.1 Data collection and processing

LASV nucleotide sequences from both S and L segments of the viral genome were obtained from the NCBI GenBank [20]. The search query run on 24 Sep 2021 was for “Lassa mammarenavirus” in the organism field of the NCBI nucleotide dataset. Data were obtained using the NCBI Entrez API with analysis conducted using the “genbankr” package within the R statistical programming language [20–22]. Associated citations were manually retrieved to identify missing metadata for sequences including hosts and geographic location of samples. Sequences with large portions (10% missing compared to reference sequences, NC_004296.1 and NC_004297.1 for S and L segments respectively) of missing nucleotide data on the L- or S-segment or lacking associated metadata (collection year, host species, country, and geographical region of sampling) were excluded from phylogenetic analysis. Nucleotide sequences were aligned using the ‘map to reference’ tool on Geneious Prime 20201.2 (https://www.geneious.com). Alignment, visual inspection and manual editing were performed, and entries that contained >100 continuous ambiguous nucleotide calls were excluded (S1 Data). We first summarise available metadata on year of collection, the species of origin of the LASV sequence and the country of sampling.

Obtained sequences are associated with the level-1 administrative region of sampling. For sequences with no sub-national location we enriched available metadata by extracting location data from associated publications. We geocoded the provided location (e.g., longitude and latitude, or town name) using the Google Geocoding API and the “ggmap” R package [23]. These produced longitude and latitude locations were associated with level-1 administrative regions using the “sf” R package, shapefiles were obtained from GADM 4.0. and accessed through the “geodata” R package [24,25]. Additional cleaned and enriched was archived on Zenodo (https://doi.org/10.5281/zenodo.6340162).

### 2.2 Sequencing bias

We compared the number of sequences returned in GenBank from each country with the number of Lassa fever (LF) cases reported from a country between 2008 and February 2023 to test the correlation between availability of sequences and reported LF cases.

The number of reported LF cases aggregated to country level was used as a measure of national LASV transmission intensity. This period was selected by the study team due to increasing standardisation of case reporting since 2008 [26,27]. Prior to 2008 cases were only sporadically reported as individual outbreaks in academic literature or outbreak reports [28]. The extension of the time-period to February 2023 (compared to the search being run in September 2021) was to incorporate more recent data from the continually improving surveillance in the endemic region. The case dataset (S2 Data) was compiled from a systematic review of the literature, public health, outbreak response and ministry of health resources. The references of data sources used to compile the reported case dataset are included in S2 Data.

We report the Pearson’s product-moment correlation coefficient (*r*), the number of degrees of freedom, the 95% confidence interval (95% C.I.) and the *p*-value for a null-hypothesis of no correlation between the number of sequences obtained from a country and the national transmission intensity of LASV. We then tested the association between the number of available sequences obtained from a) humans or b) rodent hosts with LASV transmission intensity to test the null-hypothesis that the number of both human derived and rodent derived sequences from a country was not associated with national LASV transmission intensity. The “stats” R package was used for all correlation tests [22].

To explore the bias of sequenced samples at a sub-national level the origin of a sequenced sample was geocoded using the Google Geocoding API and the “ggmap” R package [23]. Reported sequence locations were associated with level-1 administrative regions using the “sf” R package, shapefiles were obtained from GADM 4.0.2. and accessed through the “geodata” R package [24,25,29]. We stratified sequences into human and rodent sourced samples to visualise the spatial heterogeneity of sampling within rodent hosts and infected humans.

To estimate the relative sampling effort bias at the sub-national level (i.e., level-1 administrative regions), the number of samples obtained within a region was associated with the centroid of the region. As in the prior analysis, the number of reported confirmed LF cases within a region was used as a measure of regional LASV transmission intensity. To standardise this to the population size of the region, the number of reported cases within a region was divided by the human population count to produce the number of confirmed cases per 100,000 individuals. Regional population counts were obtained from WorldPop 2020 raster data accessed through the “geodata” R package [25]. GADM shapefiles of level-1 administrative regions were used to extract and aggregate human population counts from each raster cell to level-1 administrative regions using the “terra” R package [30]. The relative sequencing effort at a location is derived from the smoothed two-dimensional coefficient of a spatial Generalised Additive Model using the number of sequences as the response variable, with geographic coordinates (Longitude = X, Latitude = Y) and regional LASV transmission intensity (i.e., reported human cases per 100,000 individuals) as covariates (Eq 1). This model was constructed using the “mgcv” R package [31].

LASVsequences∼s(X*Y)+s(Reportedcasesper100,000) (1)

The smooth terms (s(X * Y) and s(Reported human cases per 100,000)) were specified as thin plate regression splines with a basis dimension (*k*) of 102 for the spatial smooth term (Longitude by Latitude) and 10 for the smooth term of LASV transmission intensity. We assessed the appropriateness of the selected basis dimensions by comparing to the estimated degrees of freedom (EDF) of the smooth terms [31]. This model is used to test the null hypothesis that there is no association between sequencing effort and LASV transmission intensity across the endemic region. We present the two-dimensional relative sequencing effort at coordinates within the study area reporting the EDF and *p-*value for the spatial smooth and LASV transmission intensity smooth terms.

### Phylogenetic analysis

Phylogenetic analysis was undertaken through Bayesian Markov Chain Monte Carlo (MCMC) method using BEAST.v1.10.4 [32]. In BEAUTi, the parameters were a substitution model as a generalised time reversible plus gamma site heterogeneity, with codon partition positions 1, 2, 3. A strict clock and a coalescent tree prior with a constant size population was used. Each analysis consisted of 20 million MCMC steps and trees were sampled every 20,000 generations. Sample collection dates from the metadata were used as tip dates to fit to a molecular clock, and country of sample collection was incorporated as a discrete state [33,34]. To assess the log files of the output TRACER.v.1.7.1 was used. Maximum-clade credibility trees were generated through TreeAnnotator v1.8.4 and visualised in FigTree.v1.4.4 [35].

## 3 Results

### 3.1 Compiled dataset

The initial dataset comprised 2,298 records (from samples obtained 1969–2019), including nucleotide sequences and associated metadata. Incomplete gene sequences and sequences lacking metadata information (n = 1,045) were removed from phylogenetic analyses. Therefore, 680 sequences of complete S segment and 573 sequences of partial L segment (L protein only) were used. Accession numbers of included and excluded sequences are available in S1 Data.

### 3.2 Descriptive analysis

**Fig 1:**
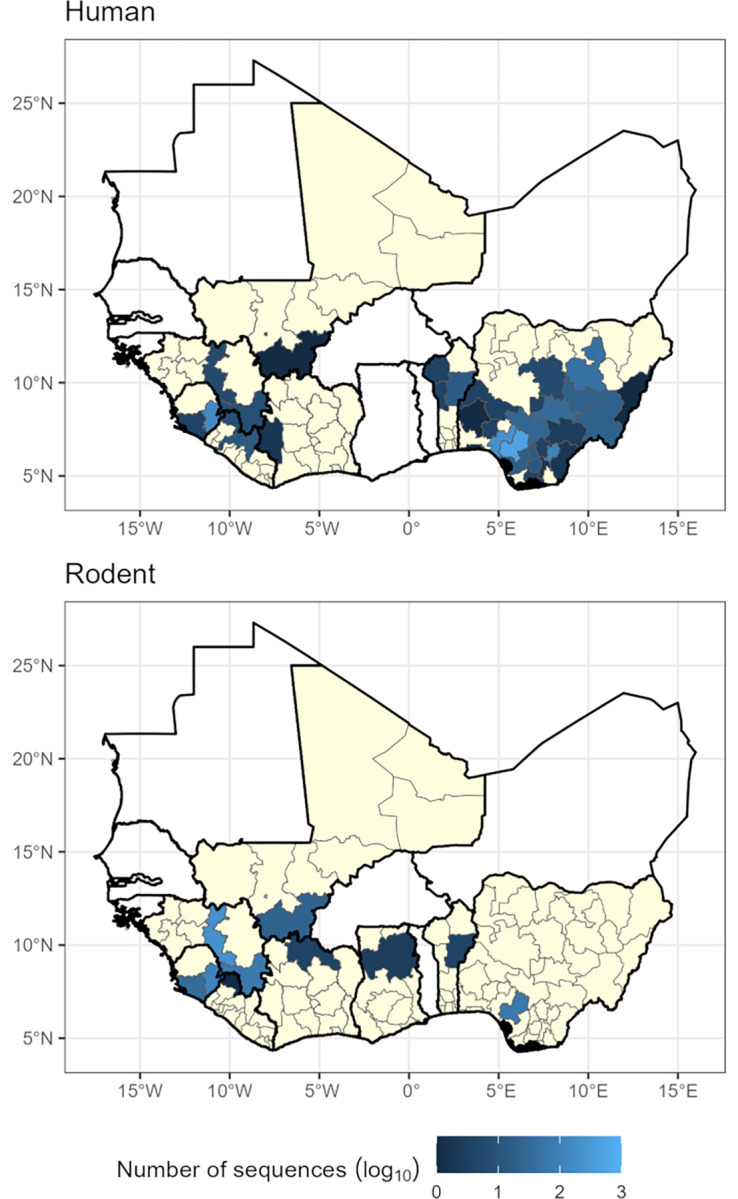
The number of sequences, shown on a log_10_ scale, retrieved from NCBI GenBank with associated sub-national sampling location and host for human samples (top, n = 1,328) and rodent samples (bottom, n = 527). Yellow regions represent level-1 administrative areas with no sequences within countries that have at least one available sequence. White countries are West African countries with no available LASV sequences. See S1 Fig for country names. Shapefiles for basemap layer obtained from GADM 4.0.2 (www.gadm.org) [29].

Year of collection was available for 2,108 records, with the oldest sequence dating from 1969 and latest from 2019. Among these records, most sequences (n = 1,936, 92%) have been obtained since 2008. Human-derived LASV sequences comprised most of the available records (67%), other host species include *Mastomys natalensis* (29%) and *Mastomys spp*. (3%), while *Mastomys erythroleucus* (n = 18), *Mus baoulei* (n = 9) and *Hylomyscus pamfi* (n = 10) represent < 1% each. The species sampled was not documented in 107 records. Country of collection was available for 2,238 records. Most sequences were produced from samples collected in Nigeria (54%), followed by Guinea (20%), Sierra Leone (14%), Liberia (4%) and Cote d’Ivoire (3%) with the remainder obtained from, Benin, Ghana, Mali and Togo (Fig 1).

Sequences for human derived samples with sub-national location data (n = 1328, 63%) were clustered in Edo State, Nigeria (n = 519, 39%), Ondo State, Nigeria (n = 220, 17%) and Eastern Province, Sierra Leone (n = 159, 12%) with 430 samples from the remaining endemic regions. Sequences from rodent samples with sub-national location data (n = 527, 25%) were most commonly obtained from Faranah, Guinea (n = 210, 39%) and Eastern Province, Sierra Leone (n = 107, 20%) with 210 samples from the regions.

### 3.3 Sequencing bias

We observed a strong positive correlation between the number of confirmed human cases between 2008–2023 and the number of GenBank deposited sequences at country level (Correlation coefficient (*r)* (degrees of freedom = 7) = 0.93, 95% Confidence Interval (95% C.I.) = 0.71–0.98, *p* < 0.001). When analysed by species source no correlation with the number of confirmed cases and the number of available rodent derived sequences was observed (*r*(6) = -0.019 95% C.I. -0.71–0.69, *p =* 0.96). There remained a strong positive correlation between the number of confirmed cases and human derived sequences (*r*(6) = 0.99, 95% C.I. 0.998–0.999, *p =* < 0.001).

**Fig 2:**
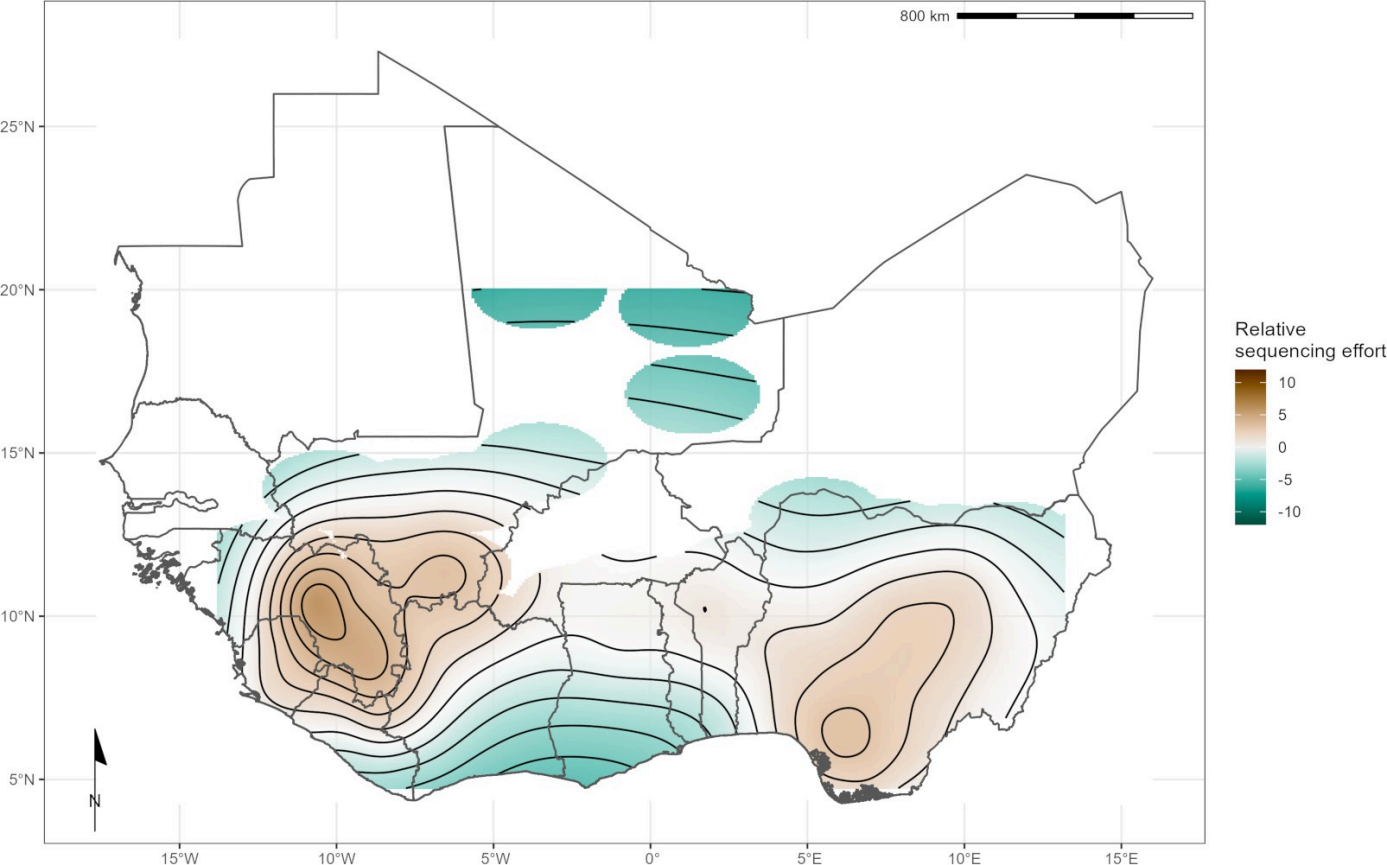
Modelled relative sequencing effort derived from both human and rodent samples. Greatest sequencing effort coincides with areas where sampling in humans (Edo, Nigeria and Kenema, Sierra Leone) and rodents (Faranah, Guinea) have historically been focussed. Shapefiles for basemap layer obtained from GADM 4.0.2 (www.gadm.org) [29].

When combining both human and rodent-derived samples at the level-1 administrative region to explore spatial sampling biases, we found differences in relative sequencing effort across the study region. The spatial smooth term identified sampling effort as greatest in Southwest Nigeria, centred over Edo State, the Faranah and Nzérékoré regions of Guinea, Eastern Province of Sierra Leone and Nimba district of Liberia (estimated degrees of freedom (EDF) = 29.2, *p* < 0.001) (Fig 2). There was a positive, non-linear association between LASV transmission intensity (measured as number of reported cases per 100,000 individuals) and the number of available rodent and human derived LASV sequences at level-1 administrative region (EDF = 3.96, p < 0.001).

### 3.4 Phylogenetic analysis

Sequences for each segment of LASV showed clustering according to previously documented lineages I-VII alongside geographical clustering with lineages I-III and VI present in Nigeria, lV in Liberia, Guinea and Sierra Leone, V in Mali and VII in Togo (S2 Fig). In this analysis only L segment sequences of lineage V from Cote d’Ivoire were included due to quality control exclusion criteria. The phylogeny of the L segment indicates an older emergence of LASV in the human population, with the most recent common ancestor (MRCA) predicted in the year 1498 in Nigeria, inference based on the S segment indicates the emergence in the year 1681 (Table 1).

**Table 1:** The most recent common ancestor (MRCA) stratified by host and country of collection of Lassa mammarenavirus (LASV) S and L segments. Samples were collected between 1969–2018.

*Host species*	*Country*	*S segment MRCA*	*L segment MRCA*
*Homo sapiens (n = 1181)*	Benin	1995	1989
	Guinea	1895	1871
	Liberia	1895	1627
	Nigeria	1681	1498
	Sierra Leone	1901	1874
	Togo	2016	2014
*Hylomyscus pamfi (n = 2)*	Nigeria	1681	1498
*Mastomys erythroleucus (n = 18)*	Guinea	1975	2010
	Nigeria	2008	2006
*Mastomys natalensis (n = 36)*	Guinea	1938	1997
	Mali	1951	2007
	Sierra Leone	1909	1979

There was a lack of sequence information from lineage I and VI, however, phylogeny suggests these lineages are basal to others in Nigeria (S2 Fig). Lineage VII in Togo is most closely related to Nigerian isolates and potentially diverged between 500–900 years ago. The divergence of lineage III and II was predicted to have occurred approximately between mid-900 and mid-1400 according to the phylogenetics trees for L and S segments respectively (S2 Fig). Introduction to countries west of Nigeria appears to be by dispersal initially to Liberia, followed by Guinea in the 1700s, followed by Sierra Leone and Mali approximately 100 years later. A lack of full segment sequences from lineage V limits calculation of divergence from the most recent common ancestor from lineage IV (approximately 200 years).

## 4 Discussion

There are several important aspects of our study and findings. First, we studied a comprehensive dataset of publicly available full-segment LASV sequences, spanning West Africa and host species, to inform our understanding of the phylogeny of LASV dispersal. Second, we identified substantial variability in the origin of available sequences and completeness of records. Third, we showed strong geographic clustering among lineages supporting prior hypotheses of radiation from both Nigeria and a subsequent introduction into Liberia [1]. Fourth, the synthesis of available metadata highlights important gaps in currently available data, including spatial bias in the sequencing of samples we propose that this should be used to inform the design of epidemiological programmes going forward.

Our analyses of 2,298 LASV sequences obtained from GenBank highlights the spatial biases in the availability of sequence data that may limit our understanding of the current and historic dispersal of LASV lineages in West Africa. First, sequence data was typically obtained from three of the eight endemic countries: Nigeria, Guinea and Sierra Leone. We found a strong association between LASV transmission intensity, measured as the number of reported human cases, and the number of available sequences (Fig 1). When stratifying by host species this trend did not remain; with rodent derived samples showing no association with LASV transmission intensity. LASV sequences obtained from rodents were relatively over-sampled from low transmission intensity settings; this may indicate a substantial lack of human case reporting in these locations. In high LASV transmission intensity settings rodents appeared to be relatively undersampled compared to human derived sequences, this is potentially an important source of bias when attempting to infer phylogeography within the reservoir host of this zoonotic pathogen. Sequence data from other countries, and more regions within them, across West Africa are required to increase confidence in the timelines of the currently inferred westward expansion. Greater focus needs to be placed on acquiring sequences from the rodent host to understand viral genetic diversity within the primary reservoir species. Comparing rodent derived sequences with those acquired from spillover into human populations may also allow identification of genetic drivers of transmission [36].

We used the number of reported cases between 2008–2023 as a measure of transmission intensity of LASV within a country over the study period. This may not be suitable due to substantial biases in the reporting of Lassa fever cases due to different surveillance sensitivity between countries and over time [3]. While unlikely, it is possible, that the magnitude of viral transmission within a country may not be directly related to the number of observed cases Therefore, in the absence of an unbiased estimate of transmission intensity we believe the number of reported cases is an acceptable proxy to model sequencing effort. We mapped the relationship between where sequences have been obtained as a function of this transmission intensity to produce a measure of relative sequencing effort to identify regions where increased LASV sequencing are required to counteract current sequencing biases (Fig 2).

Second, geographic clustering of LASV lineages, suggest isolated events of human-to-rodent transmission (S2 Fig) and the emergence of LASV dating from 1498 in Nigeria (Table 1). Similarly, Olayemi *et al*. report evidence of earlier emergence of the virus in humans than in rodents in Nigeria [34]. Comparatively limited data from non-human hosts with limited genome coverage, (69/703 sequences encompassed complete genes) produce important uncertainty around the observation of human-to-rodent transmission. Taken together, this data highlight limited surveillance among animal species, necessitating further investments in data acquisition and sharing to accurately define the spatiotemporal expansion of LASV in West Africa.

The phylogenetic analysis of LASV stratified by host species supports spatial evolution, in addition to intra-host viral evolution (S2 Fig). For instance, LASV sequences from *M*. *erytholeucus* sampled in Nigeria and Guinea clustered within lineages III and IV, respectively. Interestingly, these isolates appear to occur after the emergence of the most recent common ancestor virus circulating among humans and *M*. *natalensis* in these countries (Table 1), suggesting introduction of LASV into *M*. *erythroleucus* populations was a consequence of pathogen circulation in human and *M*. *natalensis* populations. Sequences from *M*. *natalensis* in Sierra Leone exhibit minimal clustering, and were interspersed with sequences from humans, potentially representing isolated events of pathogen introduction into human populations with spillback into commensal rodent populations (i.e., reverse zoonosis). The most recent common ancestor of LASV sequences from *M*. *natalensis* in Sierra Leone suggest a later emergence of the virus in this country. Our findings corroborate those of Olayemi et al., that within Sierra Leone LASV appears to have emerged in human hosts before rodents [34]. However, this data must be caveated by the limited information from rodent species in these locations.

There is a lower coverage of rodent-derived LASV sequences, with those from the primary reservoir *M*. *natalensis* forming fewer than one-third of all sequences (n = 642, 28%), with substantially lower sampling of other possible rodent hosts, including other *Mastomys* species. Rodent sampling has not increased at the same rate as human samples despite increased sampling effort since 2008 [15,16,37]. There is substantial heterogeneity in the locations in which rodent and human samples are available. For example, a relatively high number of rodent samples (n = 429) have been obtained from Guinea while few human sequences (n = 20) are available from these locations. The inverse is true of Nigeria where most human derived sequences are obtained (n = 1,147) but only 85 rodent sequences are available, and all of these from a single state (Edo, Nigeria). The number of suspected and reported cases was found to be positively but non-linearly associated with the number of available sequences. This is suggestive of a consolidation of research and focus of sampling in areas historically with high numbers of human cases but there remains to a paucity of sequences from elsewhere in the endemic region. The limited number of full segment sequences from rodents, from few geographic locations, limits our understanding of viral radiation in rodent hosts, particularly from species which are not considered the primary reservoir, e.g., *H*. *pamfi*. The most recent common ancestor for the viral sequence obtained from *H*. *pamfi* is estimated to be in the late 1600s; it is therefore possible lineage VI and/or *H*. *pamfi* as a reservoir of LASV has gone undetected due to lack of sufficient sampling [16].

Interpreting available LASV sequences is challenging for several reasons. A large proportion of available sequences (70%) have been obtained within Lassa fever research programs, representing spatial ascertainment bias [38–40]. In addition to these spatial biases temporal biases are apparent. Since 2016 there has been a substantial increase in the number of LASV sequences available in NCBI GenBank, reflecting increasing research effort, availability of sequencing platforms and increased data collection during Lassa fever epidemics, such as in the 2018 Nigeria Lassa fever outbreak [41–43]. There are notably fewer recorded sequences of LASV from Benin, Togo, and Ghana, suggesting a potential a gap in surveillance and research capacity in these locations or a lack of circulating LASV, despite several reported outbreaks [44–46]. Phylogenetic analysis on 60% of our initial dataset, following removal of sequences due to incompleteness or missing geographic and year of collection information (n = 1,045) demonstrated geographic clustering of LASV lineages, supporting prior analyses [16,33,34,44,47–50]. Increased data availability from Nigeria following increased LASV surveillance allowed regional analysis of phylogeny for lineages II and III supporting previous findings of expansion of these lineages from North-East Nigeria to the South-West of the country [51–53].

A substantial number (n = 869) of the sequences retrieved corresponded to short fragments (< 1 Kb) probably derived from PCR products used for diagnostic purposes rather than for viral genomic surveillance. LASV is a segmented virus, and it was not possible to identify complete genome sequences since both S and L segments are reported separately on the sequence’s repository. The molecular clock analyses from L protein indicated an earlier emergence of LASV when compared to S segment analysis (1498 and 1681 respectively), potentially because the viral RNA polymerase (L protein) is less affected by selective pressure than the S segment [11,47,54]. In arenavirus LCMV populations the number of mutations in NP and GP regions were more abundant that in the polymerase region, suggesting that mutations in the L region could be less tolerated [55]. In addition, investigations on LASV intra-host evolution, both in humans and rodents, showed that the substitution ratios varied widely across LASV genes, with the GPC gene, encoded in the S segment showing higher within-host diversity. Furthermore, this same study suggested that despite the short duration of LASV infection, B and T cells response seem to positively select escape mutations GPC [11]. Despite these challenges, this study has synthesised currently available data on LASV sequences to investigate the location and period of sampling to reconstruct the dispersal of viral lineages across the endemic region. Despite the regionalisation of LF being driven by rodent-to-human transmission, there remains scarce LASV genomic data from non-human hosts. We have mapped the locations of relative under sampling to guide targeted efforts to counteract biases in currently available data for both rodent and human derived sequences. Expanded sampling of LASV from animal species within the endemic region will improve our current understanding of LASV evolution and ecology and improve confidence in current estimates of westward expansion of Lassa fever in humans. Further understanding of the viral evolution dynamics of LASV and spatial expansion of current lineages will be vital to ensure adequate diagnostic tools are available to respond to the expected sporadic outbreaks of Lassa Fever across the region.

## Supporting information

S1 Fig Map of West Africa. displays a map of West Africa with country names for reference with Figs 1 and 2. Shapefiles for mapping obtained from GADM 4.0.2 [29].
(TIF)

S2 Fig Time-calibrated phylogeny for both the small segment (S) and large segment (L) from included LASV sequences. (TIF)

S1 Data GenBank accession number of analysed sequences. This dataset includes available data about host, country, region, year, sequence length, genome segment (L or S) and predicted MRCA.
(XLSX)

S2 Data Dataset on confirmed Lassa fever cases. This presents the number of confirmed cases of Lassa fever reported from countries between 2008 and 2023 at a subnational level that were used to calculate the number of cases per 100,000 people. References for the reports used to produce this dataset are included.
(CSV)
