# Supplementary figures and images for: Current sampling and sequencing biases of Lassa mammarenavirus limit inference from phylogeography and molecular epidemiology in Lassa fever endemic regions
Source: PLOS Glob Public Health. 2023 Nov 8;3(11):e0002159. doi: 10.1371/journal.pgph.0002159 (PMC10631635; doi:10.1371/journal.pgph.0002159)

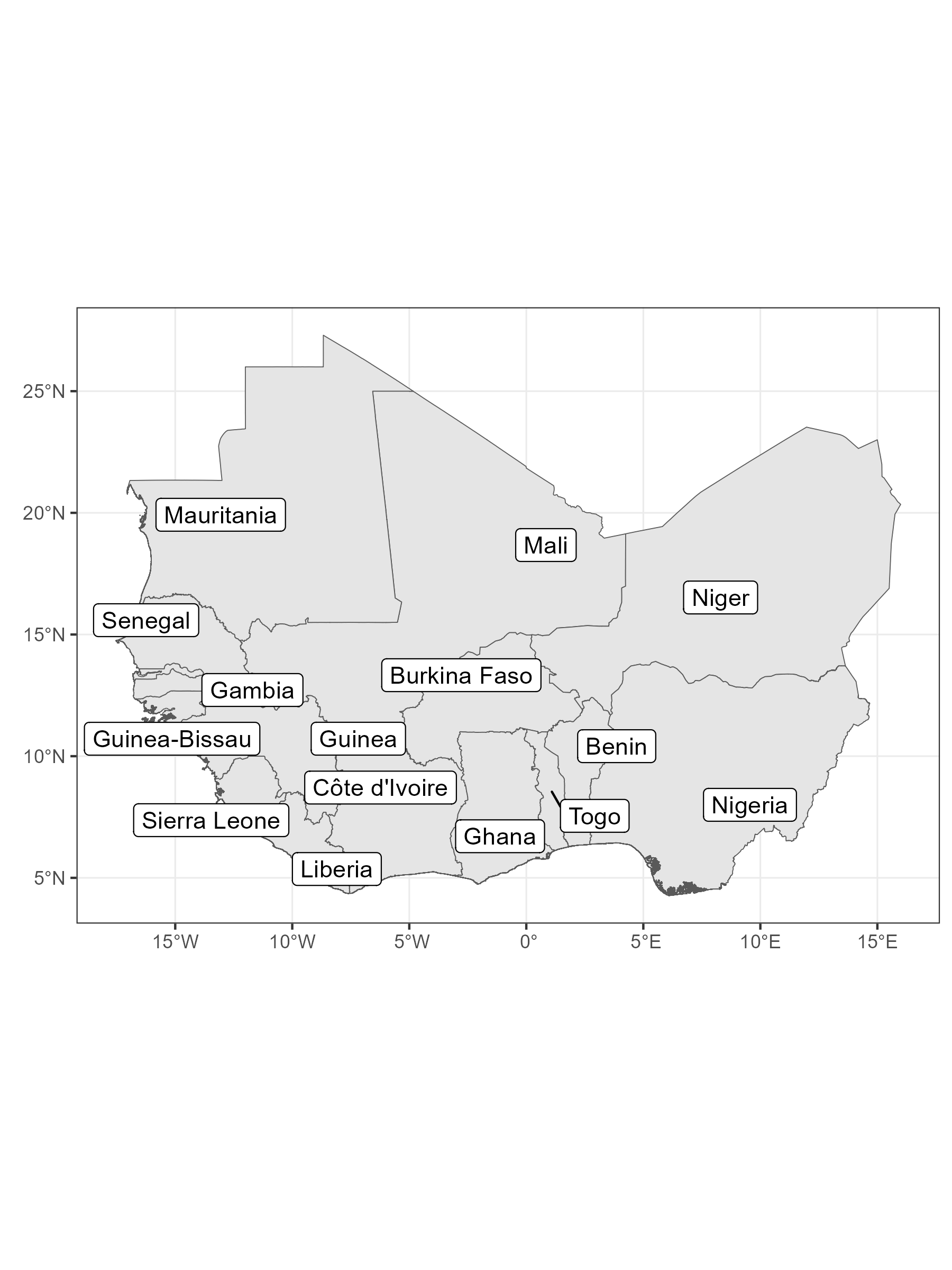

Supplement: S1 Fig — displays a map of West Africa with country names for reference with Figs 1 and 2. Shapefiles for mapping obtained from GADM 4.0.2 [29]. (TIF) [file pgph.0002159.s001.tif]

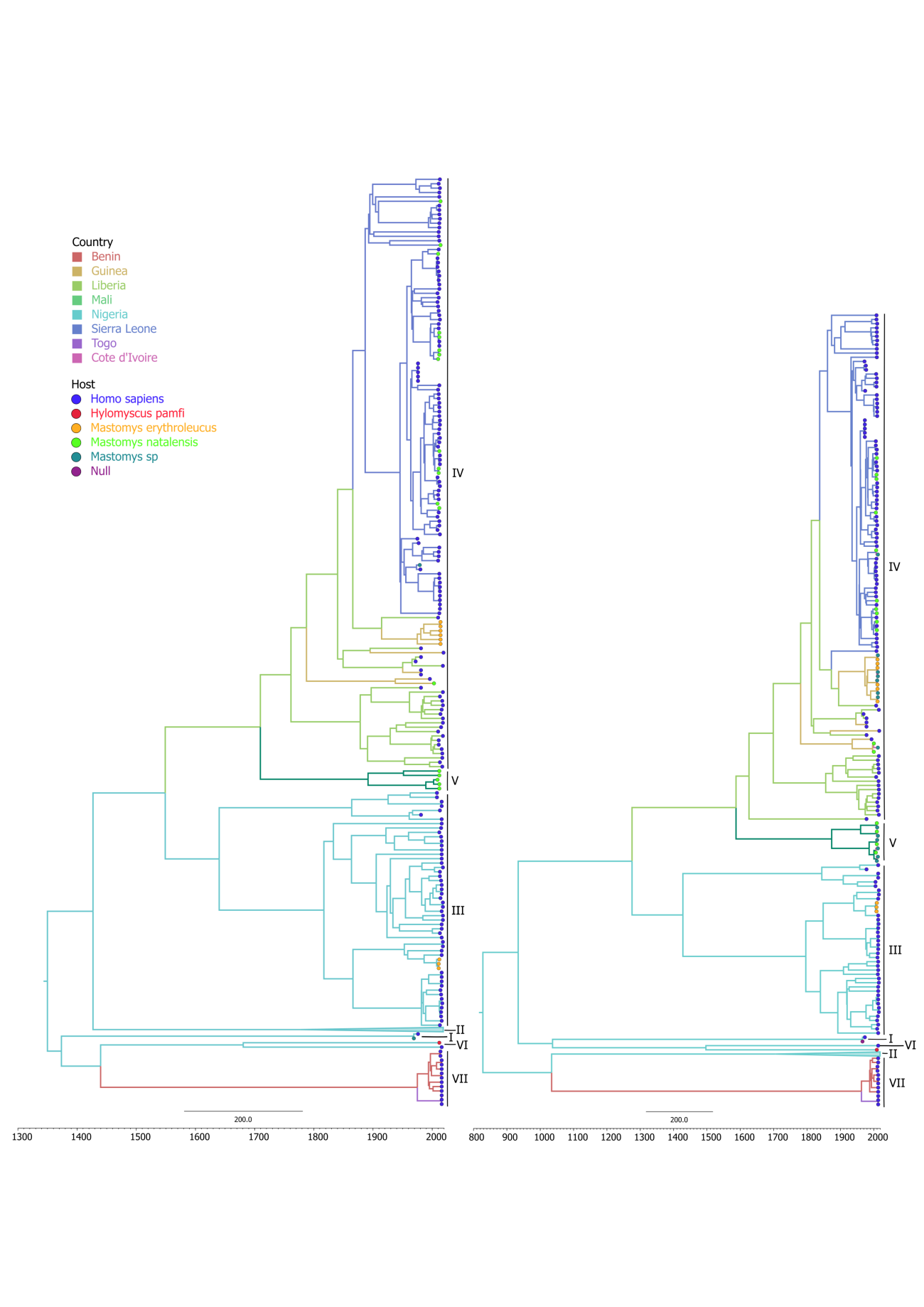

Supplement: S2 Fig — (TIF) [file pgph.0002159.s002.tif]
